# Membrane stiffness and myelin basic protein binding strength as molecular origin of multiple sclerosis

**DOI:** 10.1038/s41598-020-73671-3

**Published:** 2020-10-07

**Authors:** Benjamin Krugmann, Aurel Radulescu, Marie-Sousai Appavou, Alexandros Koutsioubas, Laura R. Stingaciu, Martin Dulle, Stephan Förster, Andreas M. Stadler

**Affiliations:** 1 Jülich Centre for Neutron Science at MLZ, Forschungszentrum Jülich GmbH, Lichtenbergstr. 1, 85748 Garching, Germany; 2 Institute of Physical Chemistry, RWTH Aachen University, Landoltweg 2, 52056 Aachen, Germany; 3 NScD, SNS, Oak Ridge National Laboratory, Oak Ridge, Tennessee 37830 United States; 4 Jülich Centre for Neutron Science (JCNS-1) and Institute for Biological Information Processing (IBI-8), Forschungszentrum Jülich GmbH, 52425 Jülich, Germany

**Keywords:** Biophysical chemistry, Multiple sclerosis, Intrinsically disordered proteins, Membrane biophysics, Membrane structure and assembly, Molecular biophysics

## Abstract

Myelin basic protein (MBP) and its interaction with lipids of the myelin sheath plays an important part in the pathology of multiple sclerosis (MS). Previous studies observed that changes in the myelin lipid composition lead to instabilities and enhanced local curvature of MBP-lipid multilayer structures. We investigated the molecular origin of the instability and found that the diseased lipid membrane has a 25% lower bending rigidity, thus destabilizing smooth $>1\,$µm curvature radius structures such as in giant unilamellar vesicles. MBP-mediated assembling of lipid bilayers proceeds in two steps, with a slow second step occurring over many days where native lipid membranes assemble into well-defined multilayer structures, whereas diseased lipid membranes form folded assemblies with high local curvature. For both native and diseased lipid mixtures we find that MBP forms dense liquid phases on top of the lipid membranes mediating attractive membrane interactions. Furthermore, we observe MBP to insert into its bilayer leaflet side in case of the diseased lipid mixture, whereas there is no insertion for the native mixture. Insertion increases the local membrane curvature, and could be caused by a decrease of the sphingomyelin content of the diseased lipid mixture. These findings can help to open a pathway to remyelination strategies.

## Introduction

Neurodegenerative diseases represent a major threat to human health. Multiple sclerosis (MS) is an incurable neurodegenerative disease leading to demyelination and axon damage in the human central nervous system (CNS). The myelin sheath is responsible for fast saltatory axonal signal conduction. Myelin damage slows down and halts nerve conduction. Strategies for remyelination suffer from the very limited knowledge of the molecular mechanism leading to myelin sheath instability and demyelination. The myelin sheath plays an important role for neuron signal conduction as it is the insulation layer, which enables fast signal transport over large distance in the white brain matter by reducing conduction losses. The saltatoric signal transduction of myelinated neurons is more than one order of magnitude faster than for unmyelinated neurons^1^. However, in demyelinating diseases like MS the myelin sheath can be damaged or even totally destroyed, which results in severe problems of signal conduction and, therefore, can lead to the multiple neuronal symptoms connected to demyelinating diseases^2^.

The myelin sheath is a multilamellar membrane wrapped around axons of neurons^3^. In the CNS this membrane is produced by oligodendrocytes. The cytoplasmic membrane leaflets of the oligodendrocytes are attached to each other by the interaction of the membrane lipids with myelin basic protein (MBP).

In literature many studies have been reported concerning the interaction of MBP with liposomes or flat oriented bilayers as model membranes (e.g.^4–10^). In the recent years particularly complex biomimetic membrane systems mimicking the myelin membrane have come into the focus of science (e.g.^11–17^). In those biomimetic systems, it is the complex lipid composition and the asymmetry of the lipid distribution between inner and outer membrane leaflets of myelin membranes that are considered. The myelin membrane is composed of a variety of different lipid molecules with various head sections and alkyl chain lengths ranging over a broad distribution including different saturation levels. Additionally, myelin membranes have a high amount of cholesterol. Myelin is a strongly anisotropic membrane and the inner (cytoplasmic) and outer (extracellular) leaflets are composed of very different lipid compositions. Therefore, more realistic model membranes mimicking the cytoplasmic or extracellular myelin membrane composition have been investigated recently^14,17–20^. In those studies, different lipid compositions have been chosen to mimick native healthy and diseased myelin membranes occuring in the experimental autoimmune encephalomyelitis (EAE) – the standard mouse model of MS. The effect of MBP on multilamellar native and diseased cytoplasmic model membranes was investigated and for the modified composition structural instabilities have been observed that are similar to in vivo conditions that occur in MS as well^14^.

In this work, we want to focus on the molecular origin of these structural instabilities in biosynthetic model membranes with native- and disease-like lipid composition and their interaction mechanism with MBP. We used model membranes mimicking the cytoplasmic membrane composition as MBP can only be found between the cytoplasmic leaflets. We observed significant differences of native and diseased myelin membranes and a different interaction mechanism with MBP emerges in both cases. Based on our results we suggest how small differences in lipid composition between the native and EAE modified myelin membranes can lead to such drastic changes of membrane stability that are presumably relevant for the MS disease in vivo.

## Results and discussion

### Membrane structure and MBP-mediated assembly

**Figure 1:**
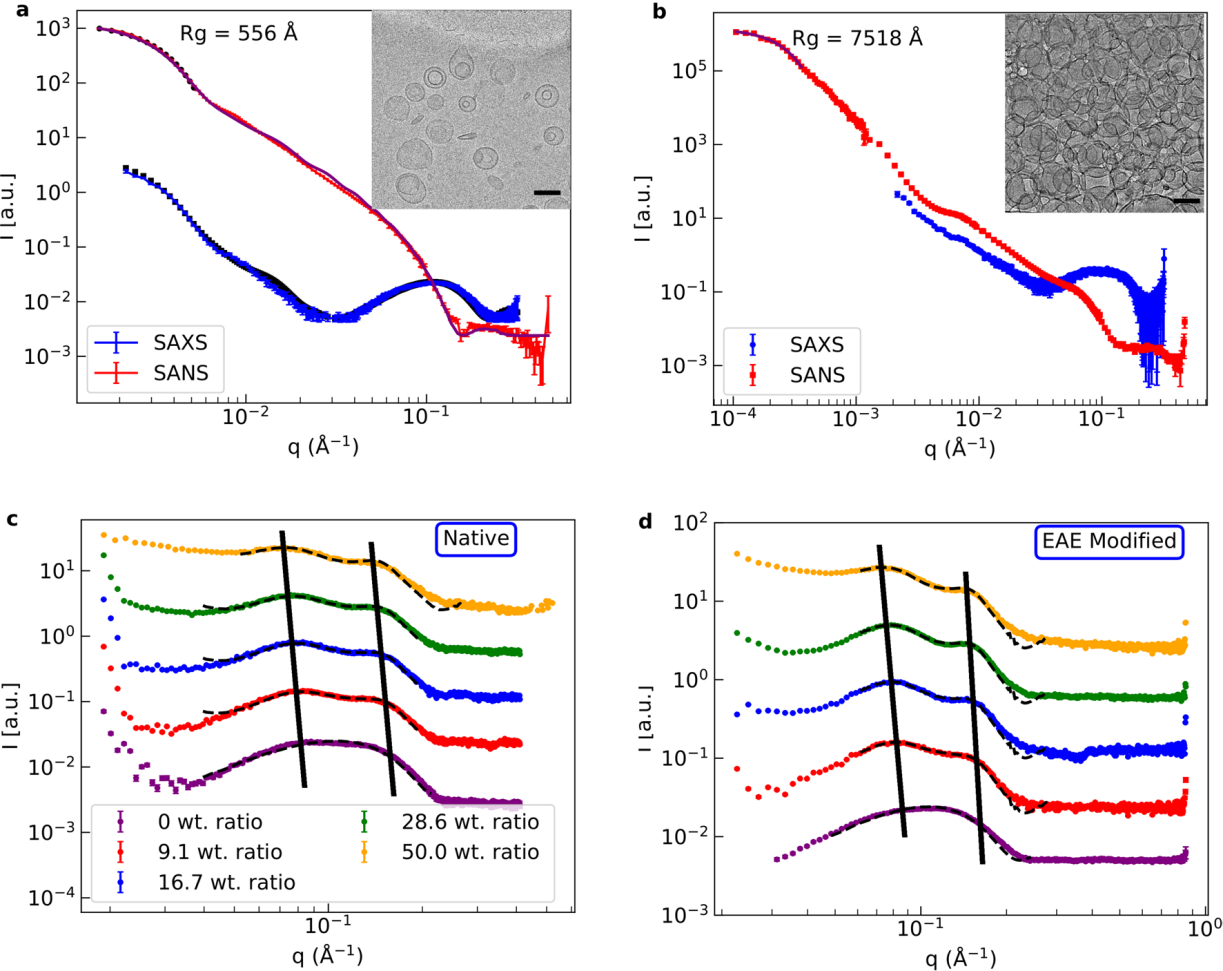
(**a**) SANS, synchrotron SAXS and cryo-TEM of native liposomes in absence of MBP. The SANS and SAXS data have been globally fitted with a simple box model. The fits are indicated by continuous lines. (**b**) SANS and synchrotron SAXS of native liposomes with 16.7 wt. ratio MBP and cryo-TEM of native liposomes with 40 wt. ratio MBP. The lipid concentration of the SANS measurements was 2.5 mg/ml, in synchrotron SAXS 5 mg/ml and in cryo-TEM 1 mg/ml. The concentration in SAXS was chosen higher for intensity reasons and the concentration of cryo-TEM lower as the blotting process increases the intensity again. The black bar in the cryo-TEM images corresponds in each case to 100 nm. In (**c**) and (**d**) SAXS data of native and EAE modified liposomes with MBP (9, 16.7, 28.6 and 50 wt. ratio) have been fitted globally by a LUV + MLV model in the region of interest. SAXS data with 0 wt. ratio MBP were fitted with the LUV model. The lipid concentrations in the SAXS measurements without MBP were 10 mg/ml and with MBP 5 mg/ml. Here, the global fit parameters of SAXS and SANS measurements of the respective lipid compositions are used for the LUV part of the model. These fits are indicated by the dashed black lines in the respective plots. For better visibility the different datasets in (**c**) and (**d**) have been vertically shifted.

In the scope of this work we have chosen to work with large unilamellar vesicles (LUV) consisting of lipid compositions as found in native or EAE modified cytoplasmic myelin since they are a well defined biomimetic model system with known geometry. The first part of our experimental study focusses on the structural properties of LUV with native and EAE-mimicking lipid composition and their aggregation process triggered by MBP. Small-angle neutron scattering (SANS) was measured on the small-angle diffractometer KWS-2^21^ and synchrotron small-angle x-ray scattering (SAXS) was recorded on the SWING beamline^22^. Cryo-transmission electron microscopy (cryo-TEM) was used in addition to obtain a direct visualisation of the investigated samples in real-space. In Fig. 1a) SANS and SAXS data as well as cryo-TEM images of vesicles with native lipid composition are shown and in Fig. S1 the respective data for EAE modified liposomes is given. In the cryo-TEM images we can see, that we obtain LUV, which sometimes entrap smaller LUV. However, the SANS and SAXS data of the vesicles can be fitted simultaneously with a simple liposome box model described in the SI 3.1. The scattering length density (SLD) value of the buffer inside the LUV seems to differ slightly from the buffer outside. This could be caused by the small vesicles that sometimes are enclosed by the larger ones as can be seen in the cryo-TEM pictures. Otherwise, we only need to fit the size distribution (radius $R \pm$ gaussian standard deviation of the size distribution *dR*) of the vesicles and the hydration (buffer penetration) *h* of the head section. Both lipid compositions roughly have the size distribution $50 \pm 15\,$nm. The obtained values of the free SAS fit parameters are compiled in Table S1 and demonstrate the monodispersity of our LUV samples for both membrane compositions with membrane properties previously identified for supported bilayers.

When MBP is added to the LUV solutions the SANS and SAXS profiles change significantly. The emergence of two correlation peaks can be observed accompanied by a shift of the Guinier regime to smaller *q*-values. These can be observed in Fig. 1b) for LUV with native lipid composition and in Fig. S1c) for modified membranes. In both cases we see an increase of the radius of gyration $R_\text {g}$ (given in figures) of more than an order of magnitude. We suspect this effect to be linked to the formation of large aggregates observed in the inset of Fig. 1b) for native membranes and in Fig. S1d) for modified membranes. Here, we added MBP with a 16.7 wt. ratio of MBP in the SAS measurements and 40 wt. ratio in the cryo-TEM. To be sure that the aggregation happens not due to crowding effects we also measured SANS of pure native and EAE modified vesicles at a high concentration of 25 mg/ml (Fig. S4). We could not observe any aggregation. We define the wt. ratio in the following as the weight percentage of the protein divided by the sum of the protein plus the lipids. Cryo-TEM pictures in real-space, see inset Fig. 1b) for native composition and Fig. S2b) for LUV with modified composition, directly show that the observed structural changes can be related to LUV aggregation. This is in accordance to the literature where aggregation of charged lipids in the presence of MBP was observed^23^. We investigated this assembly process of the LUV further by SAXS measurements in the solution state using the in-house SAXS instrument GANESHA.

As a first step, the MBP concentration was varied, while the LUV concentration was kept constant. Obtained SAXS data of LUV with native and EAE-modified lipid composition - with different wt. ratios of MBP—are shown in Fig. 1c,d). The following MBP weight ratios were added to those LUV solution: 9, 16.7, 28.6 and 50 wt. ratio. SAXS was measured after mixing. We observe an emerging peak at $q_1\sim 0.07\,{\AA }^{-1}$ which increases and is shifted slightly to smaller *q* with increasing MBP concentration. We also observe a second peak at $q_\text {2}=0.16\,{\AA }^{-1}$. The occurrence of the peaks can be explained by an inter-vesicle correlation in the LUV aggregation process, which is stronger for the modified EAE lipid composition as the correlation peaks are more pronounced in Fig. 1 for the modified membranes. The correlation is a mostly bi-lamellar correlation between the vesicles touching each other in the aggregates. The difference of rapid LUV aggregation might be due to the slightly higher amount of charged phosphatidylserine (PS) in EAE modified membranes (PS 7.4 %) as compared to the native lipid composition (PS 7.0 %). To fit these SAXS curves we modify the LUV model by adding a multilamellar vesicle (MLV) fraction:

1 $$\begin{aligned} I(q)=c + A\cdot \left[ (1-\Phi )I_\text {LUV}(q)+\Phi I_\text {MLV}\right] \end{aligned}$$

Here *I* is the respective scattering intensity, *c* is a constant background, $\Phi$ is the MLV fraction. We used a LUV + MLV model as we can see in the cryo-TEM that we have aggregation but also free vesicles in these samples. The aggregates are not really MLV but in the local vicinity of the single vesicles they touch other vesicles, so that both their bilayers form a double bilayer which is also described by the MLV model (See number of bilayers $N=2$ (Tables S2/S3)). Only a small part of the vesicles has more than 2 bilayers (Gaussian sigma *dN*  = 0.5). Those are accounted to the not perfectly unilammelar vesicles in the sample (inset Fig. 1a). If those vesicles are in the aggregate more than two bilayers can be touching each other. The model is explained in more detail in the SI 3.2. The fits are shown as dashed black lines in Fig. 1c,d). All fit parameters are summarized in Tables S2/S3.

**Figure 2:**
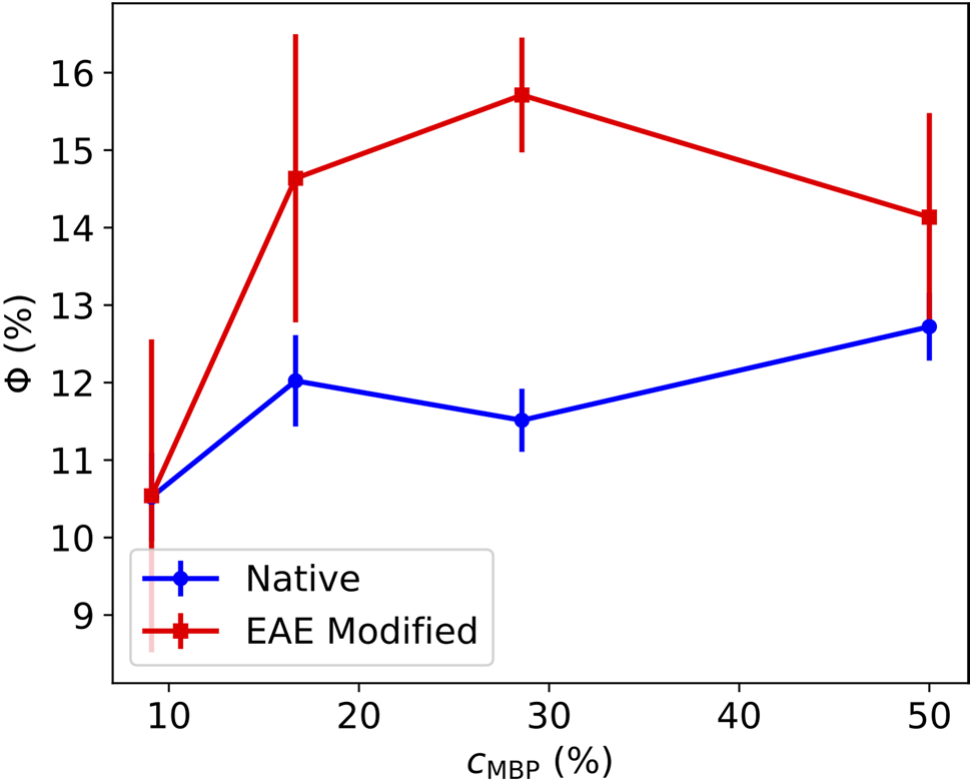
MLV fraction of the fits of native and EAE modified liposomes with MBP (9, 16.7, 28.6 and 50 wt. ratio).

**Figure 3:**
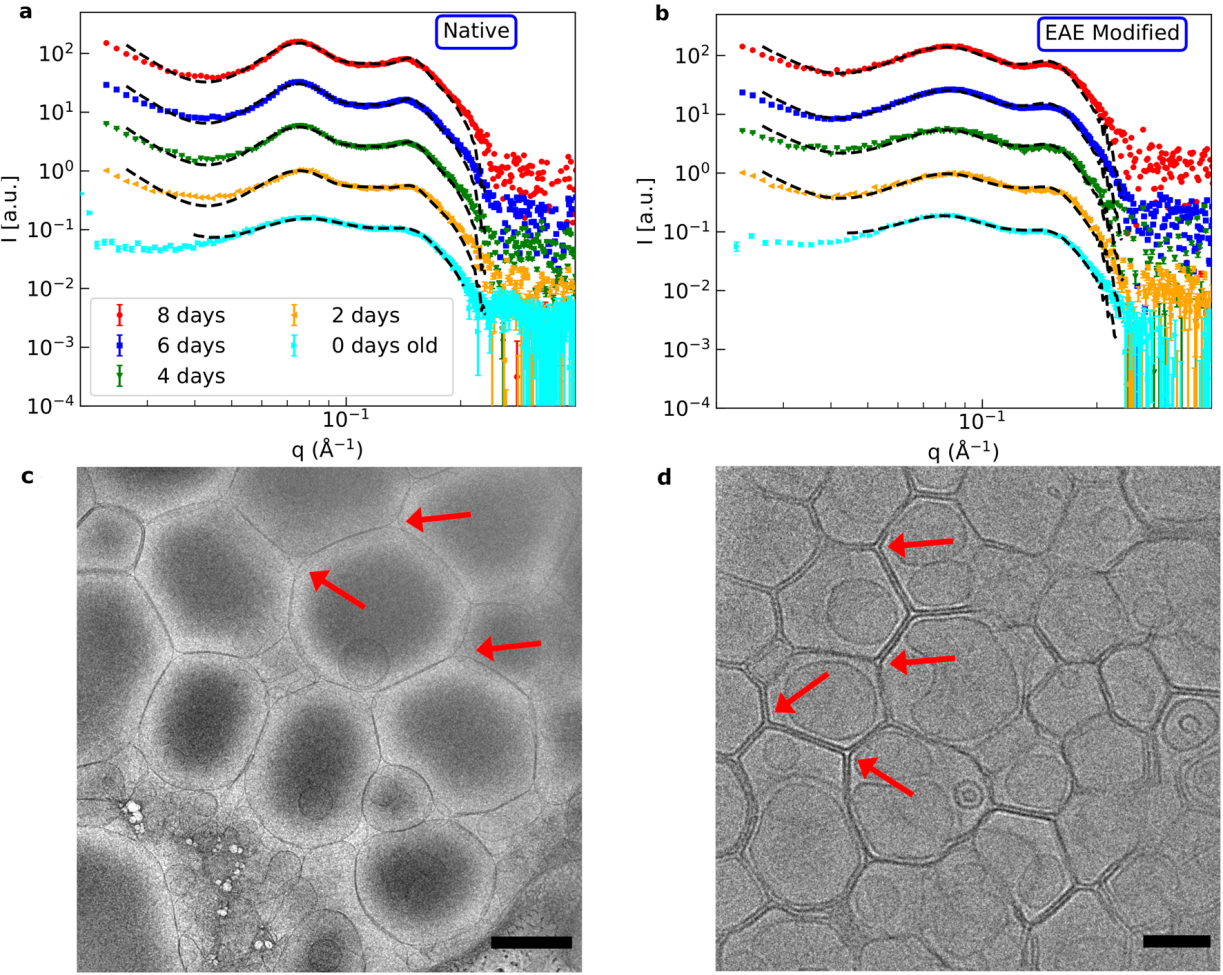
Kinetics of MBP-mediated assembly process followed by SAXS. SAXS data of (**a**) native and (**b**) EAE modified membranes mixed with 16.7 wt. ratio MBP 0, 2, 4, 6 and 8 days before the measurement. In **c)** and **d)** cryo-TEM pictures of aged native (5 days) and EAE modified (6 days) liposomes with 16.7 wt. ratio MBP are shown. The black bar in (**c**) corresponds to 500 nm and in (**d**) to 100 nm. For better visibility the different datasets in (**a**) and (**b**) have been vertically shifted. In c) some of the rounded edges and in d) some of the sharp edges have been marked by red arrows. The samples for SAXS and cryo-TEM were taken from the same batch for better comparison and had a lipid concentration of 5 mg/ml.

**Figure 4:**
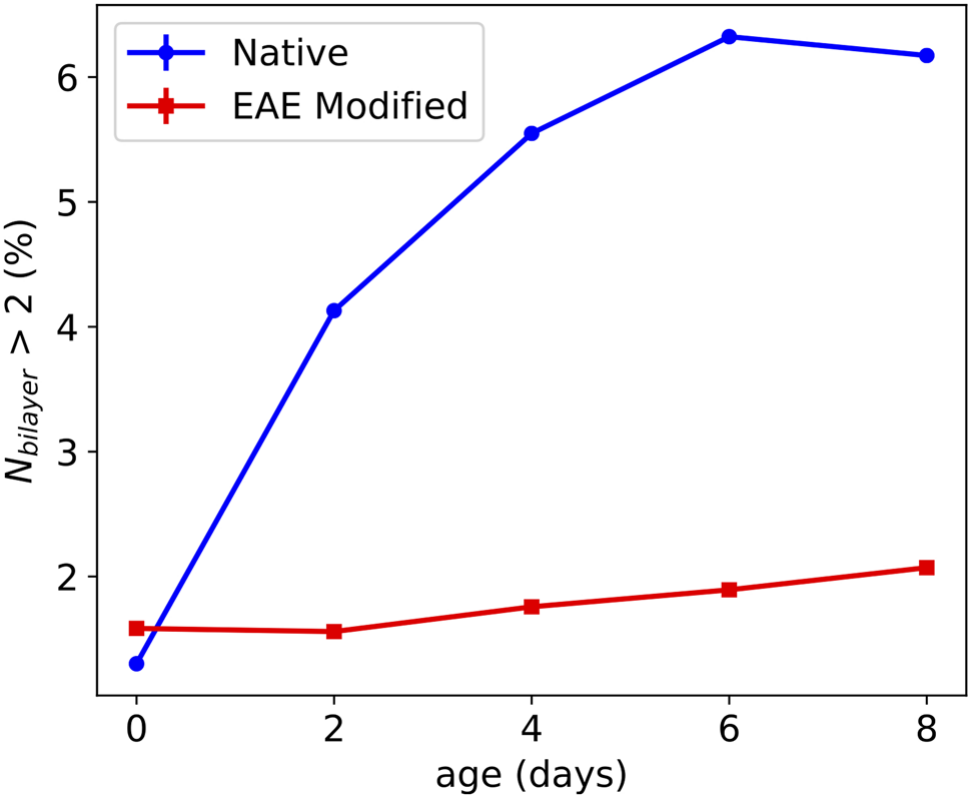
Number fraction of multilayers with a number of bilayers N > 2 in aging vesicles of native and EAE modified composition. The values have been extracted from the LUV + MLV fits shown in Fig. 3.

The thickness of the MBP-layer increases from $d_\text {MBP}=35\,{\AA }$ to $d_\text {MBP}=39\,{\AA }$ in the native case and from $d_\text {MBP}= 33.5\,{\AA }$ to $d_\text {MBP}= 37\,{\AA }$ in the EAE modified case when increasing the wt ratio from 9 to 50%. This can be explained with an increase of the amount of MBP between the bilayers with increasing MBP weight ratio. $\Phi$ is in the modified case higher than in the native one and in both cases increases with the MBP concentration, see Fig. 2.We can conclude that the rapid aggregation mechanism is similar for native and EAE modified LUV but stronger in the latter case. After the first rapid aggregation, we could identify a slower secondary process during which the organisation of the LUV changes further. The secondary process was followed with SAXS and cryo-TEM as a function of incubation time. In detail, MBP was added with a concentration of $c_\text {MBP}= 1\,$mg/ml to a LUV solution with a concentration of $c_\text {LUV}=5\,$mg/ml. Then SAXS and cryo-TEM were measured at different time points after mixture. The normalized SAXS data is shown in Fig. 3a) for native LUV and in Fig. 3b) for EAE modified liposomes. The LUV + MBP sample of the initial state (0 days) was prepared freshly just before the SAXS measurement, while samples with different incubation time (2, 4, 6 and 8 days ) were prepared ahead of the experiment. Directly after mixing, MBP leads to rapid aggregation of the vesicles. This can be seen most clearly in cryo-TEM (Fig. S2). Afterwards, further structural rearrangement occurs slowly within a few days. For the modified LUV mimicking EAE conditions no structural change is observed as the SAXS peak profile does not change with time in Fig. 3b). However, for the native liposomes (Fig. 3a) the secondary slow structural change is significantly stronger as both the first and second SAXS peak are getting more pronounced. This is an indication that the lamellar stacking is getting more regular or that more consecutive layers are formed (Fig. S3). For the fit we again utilize the LUV + MLV model described in the SI 3.2. The fit parameters are given in Tables  S4/S5. The difference between native and EAE modified lipid composition is on the one hand that $\Phi$ increases - accounting for the formation of multilamellar structure - only in the native case as the aging proceeds and on the other hand that the polydispersity *dN* increases for the native case - meaning that more membranes with a number of layers $N_\text {MLV}>2$ are in the sample which is shown in Fig. 4. In the EAE modified case $N_\text {MLV}>2$ stays stable at $\sim 2$%. However, in the native case $N_\text {MLV}>2$ clearly increases (0 days: $\sim 1.5$%, 8 days: $\sim 6$%). This is an additional indication that the secondary process only occurs for the native membranes. The cryo-TEM pictures of aged native liposomes show the formation of giant unilamellar vesicles (GUVs) with partially strongly multilamellar structures (Fig. 3c)), while the cryo-TEM of modified membranes (3 d)) shows LUVs stuck to each other but with the original vesicle diameter of around 100 nm. Therefore, structural changes probed by kinetic SAXS experiments can be identified to the formation of multilamellar structure that occurs for LUV with native membrane composition but not for EAE modified membrane composition. In this section, we showed how the liposomes of native and EAE modified lipid composition form different structures when brought in contact with MBP. As we will demonstrate in the following section, these differences can be explained to a large amount by consideration of the bending rigidity of both membranes.

### Membrane bending rigidity

**Figure 5:**
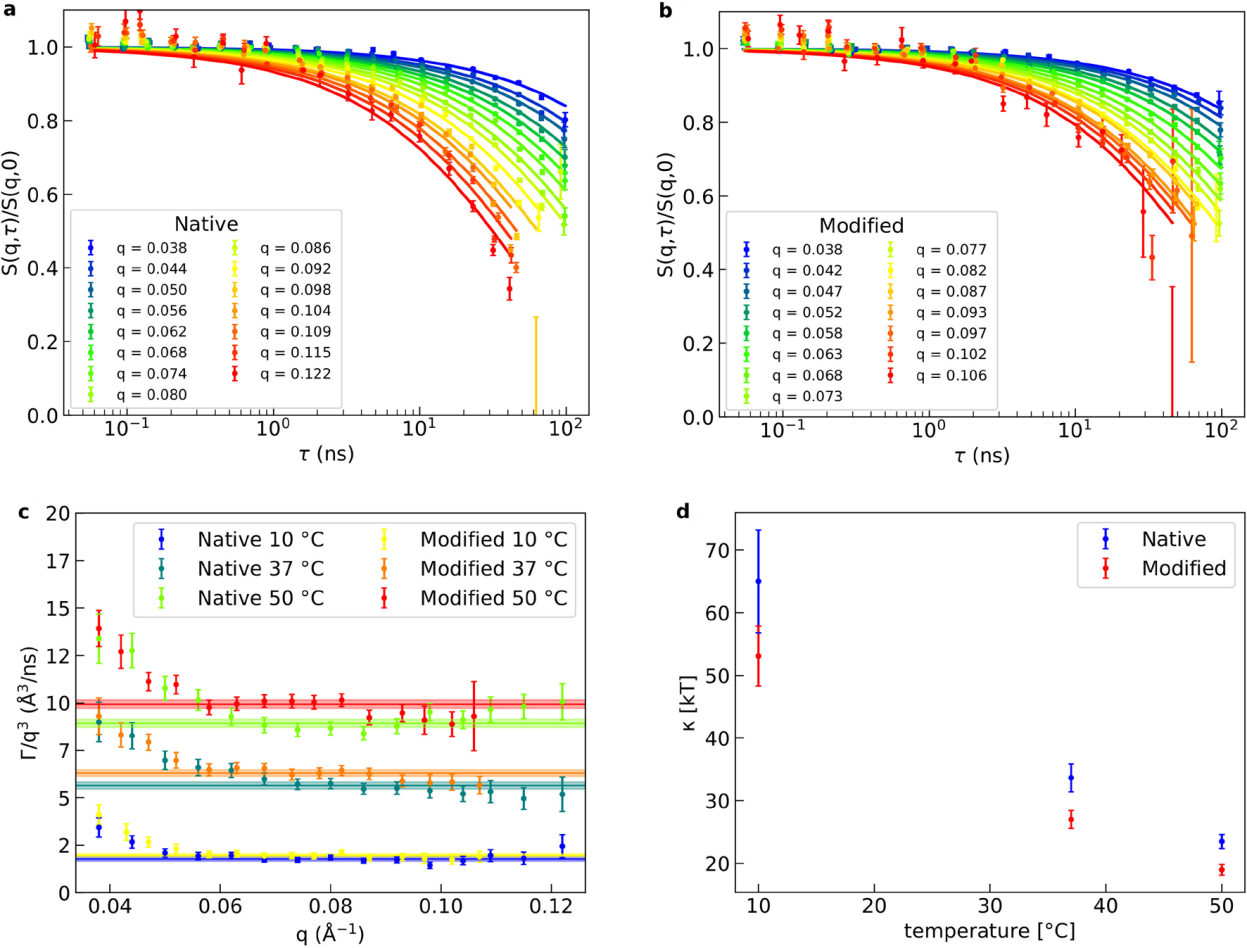
Dynamics of LUV bending fluctuations measured by NSE and determination of bending rigidity. NSE data of **(a)** native and **(b)** EAE modified liposomes at 50$\,^\circ$C. The solid lines of the respective color show the respective fit with Zilman-Granek + center of mass diffusion model for each *q*-value. In **(c)** the fit parameter $\Gamma /q^3$ is plotted against *q* and fitted with a constant for each temperature and composition. The fit is indicated as a solid line in the respective color and its standard error by the thickness of the slightly transparent enveloping lines. In **(d)** the bending rigidity $\kappa$ calculated of the $\Gamma /q^3$ fit values is plotted over the temperature for native and EAE modified lipid composition.

One important property of membranes is their elastic behavior, which is described by the Helfrich Hamiltonian^24^. This Hamiltonian is governed by the bending rigidity $\kappa$ and the spontaneous curvature $c_0$. In our case we work with bilayers with symmetrical lipid composition of cytoplasmic myelin leaflets. In this case the spontaneous curvature is negligible and the the elastic behaviour is largely governed by the bending rigidity $\kappa$. To measure the membrane bending rigidity $\kappa$ we rely on neutron spin echo (NSE) spectroscopy since it has the right time resolution to follow membrane fluctuations. NSE has demonstrated the capability to measure membrane stiffness and bending fluctuations^25–27^. NSE measurements reported here have also been performed on LUV with native and EAE modified lipid composition at a concentration of 25 mg/ml . To check unilamellarity we performed additionally SANS and SAXS measurements on exactly the same samples. Those SANS/ SAXS data are shown in Fig. S4 together with the structure factor *S*(*q*) calculated by dividing the SANS data of the NSE samples of 25 mg/ml by the SANS data of the NSE samples diluted to 2.5 mg/ml. As we can see the structure factor is in the NSE *q*-area $S(q)=1$. Therefore, it can be neglected in the following analysis. As the SANS/SAXS data can be fitted by the LUV model in the SI 3.1, we could confirm that we have unilamellar vesicles. Since liposomes of 100 nm diameter are quasi-flat on a molecular scale we can employ the Zilman-Granek model^28^ for phospholipid bilayer membrane undulations combined with center of mass diffusion.

2 $$\begin{aligned} S(q,\tau )/S(q) = A\cdot \text {exp}\left( -D_0q^2\tau \right) \text {exp}\left( -(\Gamma \tau )^{2/3}\right) . \end{aligned}$$

*A* is a scaling factor, $D_0$ is the diffusion constant, $\tau$ is the Fourier time and $\Gamma$ is the relaxation rate which is defined as the following for Zilman-Granek

3 $$\begin{aligned} \Gamma = 0.025\gamma \left( \kappa /k_\text {B}T\right) ^{-0.5}\frac{k_\text {B}T}{\eta } q^3. \end{aligned}$$

Here, $$\gamma$$ is function of $$\kappa /k_\text {B}T$$ which approaches unity for $$\kappa /k_\text {B}T>>1$$, $$\kappa$$ is the bending rigidity and $$\eta$$ the viscosity of the solvent. It has been shown that for NSE measurements the internal friction has to be considered so that $$\kappa$$ has to be replaced by $${\tilde{\kappa }}=\kappa +2d^2 k_\text {m}$$^29^. Here, *d* is the monolayer thickness and $$k_\text {m}$$ is the compressibility module of the monolayer. However, this can also be simulated roughly by a effective viscosity of $$\hbox {D}_2\hbox {O}$$$$\eta_{\text{eff}}=4\eta_{{{\text{D}}_2}{\text{O}}}$$^30,31^. Since it is our goal to compare the native and modified $$\kappa$$ and not to estimate precise values of $$\kappa$$ this approximation is reasonably good.

The diffusion coefficient is calculated by the Stokes-Einstein equation with the vesicle radius of 50 nm. An exemplary NSE measurement of each lipid composition is shown in Fig. 5a) for native and Fig. 5b) for EAE modified membranes. The respective fit is shown as solid line. We measured NSE at 3 temperatures 10$\,^\circ$C, 37$\,^\circ$C and 50$\,^\circ$C (Figs. 5 and S5). From each Zilman-Granek fit of each *q*-value we can calculate the factor $\Gamma /q^3$. When plotted against *q* this should give a constant (Fig. 5c). We can see that for small *q*-values our data deviates from a constant. This can be explained by the fact that for these values we observe almost no relaxation and therefore the fit is not reliable. Therefore, the first few *q*-values, where the values deviate strongly from a constant are neglected in the following fit. The fit of the constant area of $\Gamma /q^3$ is plotted as a solid line with an error band highlighting the standard error of those fits in Fig. 5c). From $\Gamma /q^3$ we can now calculate $\kappa$ using Eq. 3. The obtained values of the bending rigidity with error are shown in Fig. 5d). As we can see the bending rigidity decreases with the temperature and the rigidity of native membranes is continuously around 25% higher than those of the EAE modified membranes at all temperatures. From previous studies it is known that by adding cholesterol to a membrane the bending rigidiy is increased, which is not the case here (Native: 31.6 %, EAE: 37.4 % cholesterol)^25,32^. We also know that unsaturated lipids decrease the bending rigidity as they introduce disorder in the system. Therefore, the decrease of sphingomyelin in the EAE modified membrane - which is mostly saturated - should decrease its bending rigidity (Native: 6.2 %, EAE: 2.2 % sphingomyelin). In conclusion, despite of the higher amount of cholesterol in the EAE membrane, the decrease of sphingomyelin content seems to be the major driving force for the overall reduction of the bending rigidity. A higher bending rigidity can be connected to structural properties of membranes as rigid membranes try to minimize their local curvature^24^. This leads to two effects that we can observe in cryo-TEM measurements of aged LUV incubated with MBP in Fig. 3c) and 3 d). Firstly, native vesicles show the tendency to fuse and form giant vesicles with a diameter of around 1 µm while modified membranes stay at the extrusion diameter of 100 nm. Since larger vesicles have a lower curvature this is only happening for the rigid native vesicles. The EAE modified vesicles form sharp edges between 3 vesicles. This happens probably to maximise contact area between the membranes with the protein contact in between. Those edges have of course an extremely high curvature. Therefore, native membranes avoid these sharp edges and form rounded edges with buffer in the gap despite also being attracted by the positive charge of the MBP as the opposing bending force gets too strong, which results in the formation of GUV and multilamellar structure. As we have shown above the interaction of MBP with even such a simple system as biomimetic vesicles is quite complicated and leads to heterogeneous structures. To understand the basic interaction of the protein with the membrane lipids we focus in the next section on the simpler geometry of the supported bilayer.

### MBP membrane insertion

**Figure 6:**
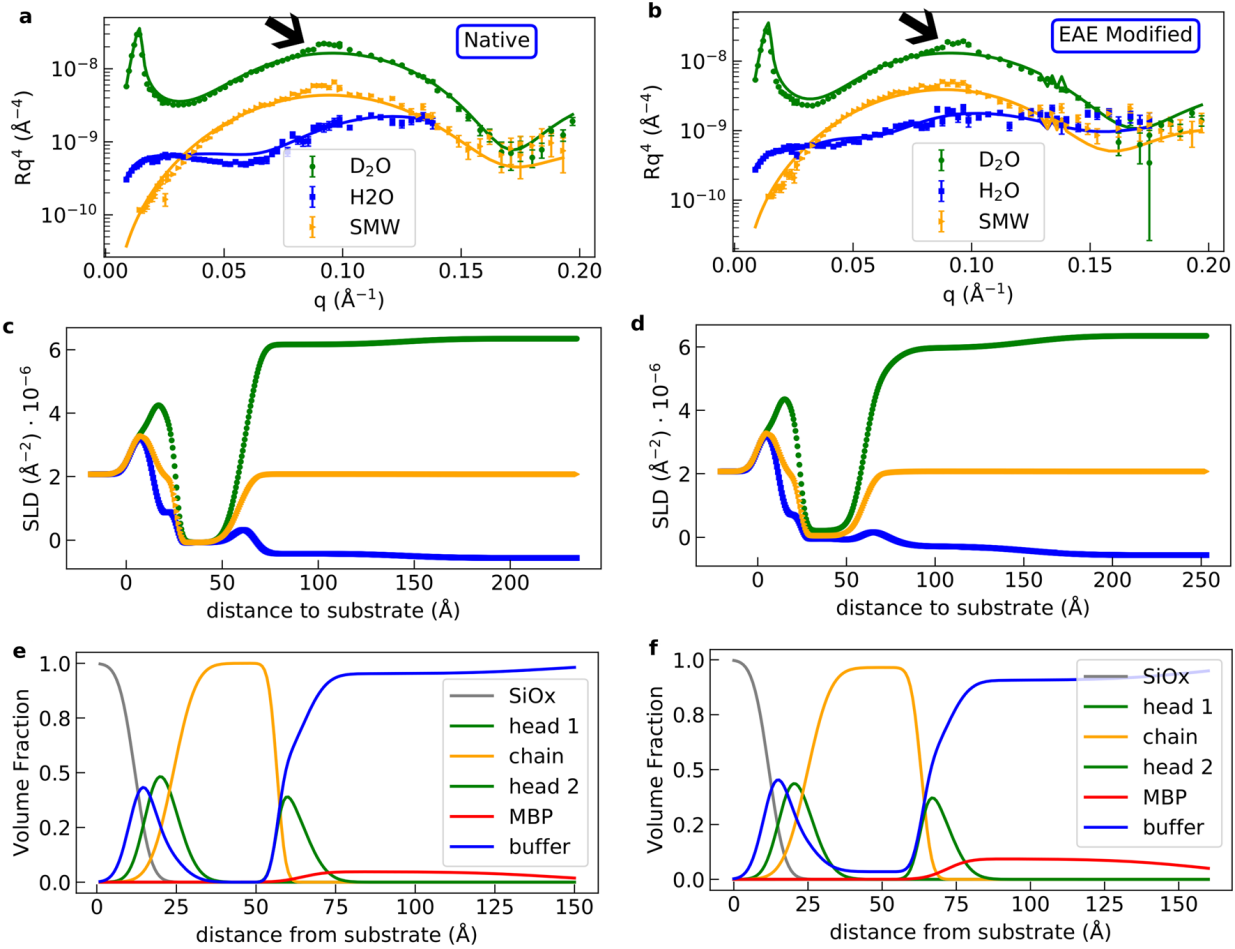
Determination of membrane profile of oriented bilayers by NR. NR data of **(a)** native and **(b)** EAE modified membranes with MBP. Each dataset with the 3 contrasts $\hbox {D}_2\hbox {O}$, $\hbox {H}_2\hbox {O}$ and silicon matched water (SMW) have been fitted globally. These fits are indicated by the solid lines in the respective color. Furthermore, the SLD profile of **c)** native and **d)** EAE modified membranes with MBP, which is calculated from the fits, is shown. The volume fractions over the distance from the different parts of the membrane are shown **e)** for the native membrane and **f)** for the EAE modified membrane.

**Table 1:** Thicknesses $d_\text {i}$ and hydration (buffer penetration) $h_\text {i}$ of head, chain and MBP sections of native and EAE modified supported bilayers measured by neutron reflectometry with and without MBP. Here, i stands for head, chain or MBP.

	nat	nat $+$ MBP	EAE	EAE $+$ MBP
$d_\text {head}$ ($\AA$)	8.0±0.4	8.0±0.4	8.1±0.4	8.1±0.4
$d_\text {chain}$ ($\AA$)	33±2	32.6±0.2	31±2	35.8±0.3
$d_\text {MBP}$ ($\AA$)	0	84±3	0	80±5
$h_\text {head,inner}$ (%)	31±2	25±2	33±2	31±5
$h_\text {chain}$ (%)	0	0	0	3.5±1.1
$h_\text {head,outer}$ (%)	31±2	47±1	33±2	48±1
$h_\text {MBP}$ (%)	0	95±1	0	91±2

In form of a supported bilayer the membrane is fixed to a substrate and, therefore, no aggregation effects like in the measurements using LUV can occur. This allows a precise measurement of the vertical membrane layer structure by neutron reflectometry (NR), which is very sensitive for changes induced by MBP binding. In particular, how MBP binding differs for both lipid compositions. The NR method has been used before to investigate interaction of membranes with protein or peptides, see e.g.^20,33–35^. NR was measured for both native and EAE modified membrane compositions at the neutron reflectometer MARIA^36^. To form a supported bilayer on the silicon substrate the so-called vesicle fusion is utilized. In this process the vesicles fuse on the strongly hydrophilic SiO_x_ surface of the substrate and form a continuous bilayer. To make this process happen we inject vesicles in the liquid cell with the silicon substrate and wait for 15 minutes at a temperature of 50$\,^\circ$C. The so-formed supported bilayers were measured at the three different contrasts $\hbox {H}_2\hbox {O}$, $\hbox {D}_2\hbox {O}$ and silicon matched water (SMW) (38% $\hbox {D}_2\hbox {O}$) (Fig. S6). These NR curves do not differ in the precision of our measurements between the bilayers with native and EAE modified lipid composition. This is not surprising since both membranes have a similar composition with a large amount of cholesterol, which should stabilize an ordered liquid phase^37^. They could be fitted with a simple stratified layer system with the layer order Si/$\hbox {SiO}_{\text{x}}$/buffer/head/2$\cdot$chains/head/buffer using the Abeles matrix formalism. All fits have been performed with motofit^38^. In the following, MBP was added and again NR at the same contrasts was measured. The NR curves in $Rq^4$ vs *q* and the SLD profiles of native (Fig. 6a,c,e)) and EAE modified (Fig. 6b,d,f)) show a quite different behaviour when MBP is added and incubated for 15 minutes before flushing the liquid cell with buffer (Fig. S7). The NR curves with protein at a concentration of 0.1 mg/ml were fitted simultaneously with a simple stratified layer membrane model as shown before but with an additional MBP layer on top of the membrane. Therefore, the resulting layer order is Si/$\hbox {SiO}_{\text{x}}$/buffer/head/2$\cdot$chains/head/MBP/buffer. The fit results are given in Table 1. The density profile of the native membrane with bound MBP is similar to the membrane without protein. A $d_\text {MBP}\sim 80\,{\AA }$ MBP layer is formed on top and there seems to be almost no protein and buffer insertion in the membrane. The thickness of the MBP layer $d_\text {MBP,NR}\sim 80\,{\AA }$ is larger than the thickness of the MBP layers measured between the membranes in our SAXS measurement of multilamellar vesicels ($d_\text {MBP,SAXS}\sim 30\,{\AA }$). This is in accordance to the $d_\text {MBP,in\,vivo}=30\,{\AA }$ observed in fresh mammalian myelin sheath^39^ and with the MBP-layer thickness observed in similar model systems^14^ but still smaller than the maximal length of a fully disordered MBP molecule^20^. To explain this, two models have to be considered which have already been suggested in literature^20,40^. One possibility is that the proteins fold at the side attached to the membrane, but stay disordered on the other side leading to a brush-like structure^20^. The second possibility is that further MBP molecules are attached to the first folded protein stack to build up the thicker protein layer^40–43^. This can be explained by a mechanism similar to a liquid-liquid phase separation: The process is initiated by folding and attachment of the MBP molecules in direct contact with the membrane and further growth due to MBP binding. Here, the trigger is a heterotypic interaction of MBP with the negatively charged membranes. Due to the higher local protein concentration, further MBP layers can be added yielding the concentrated top layer via homotypic MBP interactions. However, further experiments with MBP and interaction partners are needed to differentiate between those models. For the EAE modified membrane, however, we can fit the data only by adding buffer insertion and swelling of the membrane ($h_\text {chain}$ = 3.5%) and also a roughly $80\,{\AA }$ MBP layer on top. The $d_\text {MBP}\sim 80\,{\AA }$ thickness of this layer is very similar to the layer thickness measured before for MBP on DMPC:DMPG membranes^20^. To explain these results we argue that it is necessary for the MBP to partially penetrate the chain section of the layer due to a stronger MBP-EAE membrane interaction than that it is the case for the native membrane. Therefore, water can enter the hydrophobic membrane section. In addition, holes might be formed in the bilayer by partial folding of lipids around the MBP molecules in their vicinity which could lead to destabilization of the EAE membrane bilayer. We also observe a small peak at $q=0.095\,{\AA }^{-1}$ for both membranes (marked by black arrows in Fig. 6a,b)). We suspect that this is the lamellar peak which is also caused by folding of the negatively charged lipid bilayer around the positively charged MBP. This effect also seems to be stronger for EAE modified membranes, which is reasonable since the MBP concentration on top of the modified membrane measured with NR seems to be substancially higher leading to water insertion in the membrane. With the results of the fitted NR data given in Table 1 we can calculate the surface excess $\Gamma$ and area per protein $A_\text {p}$ for both lipid compositions. From^44^ we know that:

4 $$\begin{aligned} \Gamma =\Phi _\text {p}\cdot \tau \cdot \rho _\text {p}^{'}. \end{aligned}$$

Here $\Phi _\text {p}$ is the volume fraction of the protein which is $1-h$, $\tau$ is the thickness of the protein layer and $\rho _\text {p}^{'}=M/V$ is the protein density. The molecular mass of the bovine MBP isoform is $M=18.4\,$kDa and the molecular volume is roughly $V=36\,\text {nm}^3$^45^. For MBP $\rho _\text {MBP}^{'}=0.51\,\text {Da}/{\AA }^3$. We can now calculate the area per protein:

5 $$\begin{aligned} A_\text {p}=\frac{M}{\Gamma }. \end{aligned}$$

The values for native and modified membranes are ${\Gamma _\text {nat}=0.356\pm 0.071\,\text {mg}/\text {m}^2}$, ${\Gamma _\text {EAE}=0.61\pm 0.14\,\text {mg}/\text {m}^2}$, ${A_\text {p,nat}=(8.6\pm 1.7)\cdot 10^3{\AA }^2}$ and ${A_\text {p,EAE}=(5.0\pm 1.5)\cdot 10^3{\AA }^2}$. The NR data of oriented bilayers revealed differences in the interactions of both lipid compositions with MBP and the formation of a concentrated roughly 80 Å thick MBP layer on top. For the EAE membrane the surface excess of MBP is twice as high (0.61 mg/m$^2$) indicating stronger MBP binding as compared to native layers (0.36 mg/m$^2$). Our experimental NR results identify strong MBP binding of biomimetic EAE model membranes as molecular origin that prevents formation of multilamellar structures of LUV and, thus, might impede formation of well-ordered myelin sheaths in MS disease.

## Conclusions

We investigated the interaction of MBP with LUV having native and EAE modified lipid compositions and the resulting assembly process of the liposomes. Binding of MBP to LUV with modified EAE membranes was found to be stronger than for native membranes leading to a higher tendency for aggregation of EAE-mimicking LUV as compared to native-like liposomes. While for EAE modified membranes the vesicles remain stable in this aggregated state, native vesicles undergo a secondary reordering process in the time range of several days during which LUV fuse and significant amount of multilamellar structure is formed. Investigations on the bending fluctuations of the LUV identified a higher bending rigidity of liposomes with native lipid composition than for EAE modified membranes. Further experiments have been performed with oriented bilayers. Here, for both lipid compositions a concentrated protein layer is formed on top of the membranes. The surface excess of MBP on top of a membrane with EAE lipid composition is higher than for the native lipid composition showing stronger MBP interactions with diseased membranes that result in structural perturbation of the membranes - which manifests itself in swelling of the membrane and in buffer insertion in the hydrocarbon-section of the membrane. This strong MBP-membrane interaction in combination with the low bending rigidity can be identified as a molecular trigger, which destabilizes EAE modified membranes and prevents the formation of multilamellar structure. We suggest that this mechanism acting on the molecular scale might prevent the generation of myelin sheath-like membrane systems under in vivo conditions as well. Our studies provide a molecular explanation of the destabilisation of the membrane structure observed in MS mouse model system EAE. However, not only modification of lipid composition, but also properties of the protein component - such as by e.g. posttranslational modifications of MBP - will play here a role. The relevance of modified physico-chemical properties of MBP needs to be investigated in more detail in future studies to understand the interplay of lipid and MBP for the MS disease.

## Materials and methods

Detailed information about Materials and Methods is available in the SI 2.

### Materials

Porcine brain lipids and ovine cholesterol was purchased from Avanti Polar Lipids (Alabaster, AL, USA) and bovine MBP from Sigma-Aldrich (St. Louis, MO, USA). Liposomes of native and EAE modified lipid composition have been prepared via extrusion through a membrane with 100 nm diameter pores. In Table S6 the lipid compositions of the two membrane types are depicted. To calculate scattering curves of the native and EAE membranes averaged x-ray/neutron scattering length densities (SLDs) of the head and chain sections were used (averaged SLD values given in Table S7). For the calculation of these average SLDs the fatty chain length composition of the different lipid molecules (see Table S8) was considered. Molar volumes $V_\text {m}$, coherent neutron scattering lengths $b_\text {coh}$ of their moieties and the neutron and x-ray SLDs of the individual head groups and chain components are given in Table S9.

### Small angle X-ray scattering

The measurements at the SWING beamline at SOLEIL^22^ and in-house SAXS measurements at the GANESHA setup were performed in standard quartz capillaries at room temperature.

### Small angle neutron scattering

The measurements at the KWS-2 diffractometer^21^ were performed at different sample-detector distances of 2 m to 20 m. Additionally, measurements with neutron focussing lenses have been performed for the very low *q*-values.

### Cryo-TEM

Samples were cryo-fixed in − 180 °C liquid ethane and carried out at temperatures of around − 180 °C. The transmission electron microscope was operated at an acceleration voltage of 200 kV. Zero-loss electron energy filtered images were taken under reduced dose conditions ($< 10000\,$e/nm ).

### Neutron reflectometry

The measurements at the MARIA neutron diffractometer^36^ were performed at two neutron wavelengths to cover the relevant *q*-range. The change of solvent contrast in the liquid cells was performed using a combination of valves and a peristaltic pump, at small flow rates of 0.5 ml/min.

### Neutron spin-echo spectroscopy

Measurements were carried out at the SNS-NSE spectrometer^46^. The NSE spectra were collected at 10 °C–50 °C for samples in 4 mm-path quartz cells, accessing a dynamical range between $0.1 \leqslant \tau _\text {max} \leqslant 130\,ns$.

## Supplementary information

Supplementary Information.

## Abbreviations

MBP: Myelin basic protein
MS: Multiple sclerosis
CNS: Central nervous system
EAE: Experimental autoimmune encephalomyelitis
LUV: Large unilamellar vesicle
CNS: Central nervous system
SANS: Small-angle neutron scattering
SAXS: Small-angle x-ray scattering
cryo-TEM: Cryo-transmission electron microscopy
SLD: Scattering length density
PS: Phosphatidylserine
MLV: Multilamellar vesicle
GUV: Giant unilamellar vesicle
NSE: Neutron spin echo
NR: Neutron reflectometry
SMW: Silicon matched water
