# Supplementary material for: Membrane stiffness and myelin basic protein binding strength as molecular origin of multiple sclerosis
Source: Sci Rep. 2020 Oct 7;10:16691. doi: 10.1038/s41598-020-73671-3 (PMC7542173; doi:10.1038/s41598-020-73671-3)
Supplement: Supplementary file 1 — Supplementary Information. [file 41598_2020_73671_MOESM1_ESM.pdf]

Supplementary Information: Membrane stiffness and myelin basic protein binding strength as molecular origin of multiple sclerosis

Benjamin Krugmann[a,b], Aurel Radulescu[a], Marie-Sousai Appavou[a], Alexandros Koutsioubas[a], Laura R. Stingaciu[c], Martin Dulle[d], Stephan Förster[d], and Andreas M. Stadler[b,d,*]

[a]Jülich Centre for Neutron Science at MLZ, Forschungszentrum Jülich GmbH, Lichtenbergstr. 1, 85748 Garching, Germany
[b]RWTH Aachen University, Institute of Physical Chemistry , Landoltweg 2, 52056 Aachen, Germany
[c]NScD, SNS, Oak Ridge National Laboratory, Oak Ridge, Tennessee 37830, United States
[d]Jülich Centre for Neutron Science (JCNS-1) and Institute for Biological Information Processing (IBI-8), Forschungszentrum Jülich GmbH, 52425 Jülich, Germany
[*] *a.stadler@fz-juelich.de*

Contents

1 Supplementary Tables and Figures **2**

2 Materials and Methods **7**
2.1 Materials 7
2.2 Lipid Composition 7
2.3 Liposome Preparation 7
2.4 Small-Angle X-Ray Scattering 9
2.5 Small-Angle Neutron Scattering 9
2.6 Cryo-TEM 10
2.7 Neutron Reflectometry 10
2.8 NR sample preparation 10
2.9 Neutron Spin Echo Spectroscopy 11

3 Small Angle Scattering Fitting Models **11**
3.1 LUV model 11
3.2 MLV model 11

1 Supplementary Tables and Figures

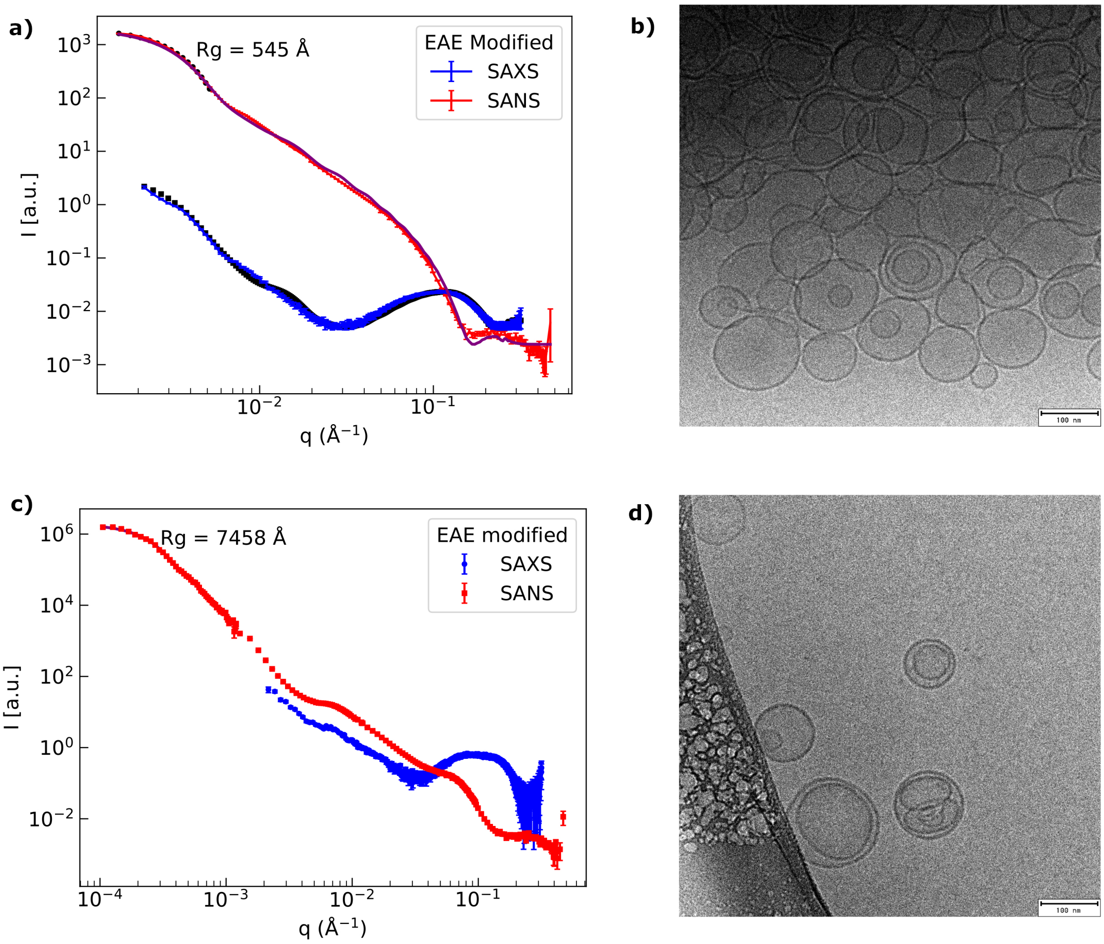

Figure S1: a) SANS, synchrotron SAXS and b) cryo-TEM of EAE modified liposomes. The SANS and SAXS data have been globally fitted with a simple box model. The fits are indicated by continuous lines. c) SANS, synchrotron SAXS and d) cryo-TEM of EAE modified liposomes with MBP added (16.7 wt.% for SAS and 25 wt.% for cryo-TEM). The lipid concentration in SANS has been 2.5 mg/ml, in SAXS 5 mg/ml and in cryo-TEM b) 1 mg/ml and in d) 1.5 mg/ml.

Table S1: Outer radius of the vesicles, width of the outer radius distribution dR, scattering length density difference between buffer inside and outside of the vesicles $\rho_{\mathrm{buffer,i}} - \rho_{\mathrm{buffer,o}}$ and water penetration of the head sections h. The values were fitted by a global fit of vesicles measured by SANS and synchrotron SAXS. The fit was performed with jscatter [1]. The errors are estimated by the fit

parameter	native neutron	native x-ray	EAE neutron	EAE x-ray
Radius (nm)	52.31±0.18	54.7±1.5	50.60±0.20	54.91±0.62
dR (nm)	13.85±0.11	14.89±0.92	13.53±0.11	16.55±0.36
$\rho_{\mathrm{buffer,i}} - \rho_{\mathrm{buffer,o}}$ ($\cdot 10^{-6}$Å^{-2})	0.15	-0.007	0.16	-0.007
h (%)	37.79±0.38	37.79±0.38	38.88 ±0.27	38.88 ±0.27

Table S2: Fit parameters of LUV + MLV fit of native liposomes MBP concentration series. Φ is the fraction of MLV. For the SLD of the MBP layer ρ_{MBP} no error is given since the fit error was extremely small. For the number of bilayers N, the Gaussian standard deviation of the number of bilayers dN and the thickness of the chain section d_{chain}

	nat + 9% MBP	nat + 17% MBP	nat + 29% MBP	nat + 50% MBP
Φ (%)	10.52 ± 0.57	12.03±0.59	11.51±0.40	12.72±0.43
ρ_{MBP} (10^{-5} Å^{-2})	1.12	1.13	1.16	1.18
d_{MBP} (Å)	35	36	37	39
N	2	2	2	2
dN	0.5	0.5	0.5	0.5
d_{chain} (Å)	31.384 ± 0.050	31.384 ± 0.050	31.384 ± 0.050	31.384 ± 0.050

Table S3: Fit parameters of LUV + MLV fit of EAE modified liposomes MBP concentration series. Φ is the fraction of MLV. For the SLD of the MBP layer ρ_{MBP} no error is given since the fit error was extremely small. For the number of bilayers N, the Gaussian standard deviation of the number of bilayers dN and the thickness of the chain section d_{chain} no error is given since those values were fixed

	EAE + 9% MBP	EAE + 17% MBP	EAE + 29% MBP	EAE + 50% MBP
Φ (%)	10.5±2.0	14.6±1.9	15.71±0.74	14.1 ± 1.3
ρ_{MBP} (10^{-5} Å^{-2})	1.13	1.12	1.13	1.18
d_{MBP} (Å)	33.5	34.5	35	37
N	2	2	2	2
dN	0.5	0.5	0.5	0.5
d_{chain} (Å)	31	31	31	31

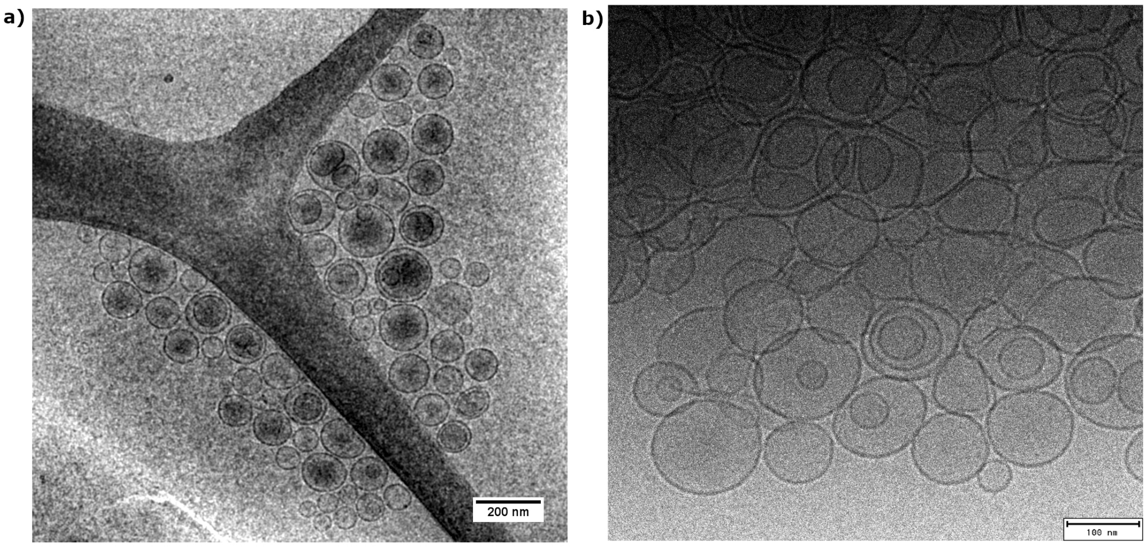

Figure S2: Cryo-TEM pictures of **a)** native and **b)** EAE modified liposomes directly after mixing with 25 wt. ratio MBP. The lipid concentration in both images was 1.5 mg/ml.

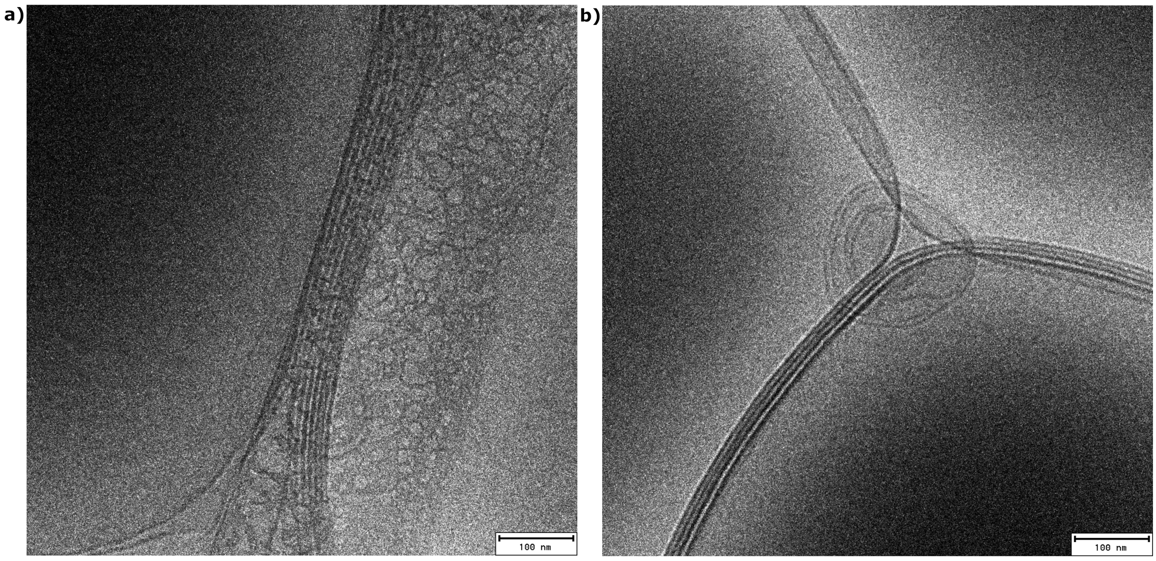

Figure S3: cryo-TEM pictures of native liposomes 5 days after mixing with 16.7 wt. ratio MBP. The lipid concentration in both images was 5 mg/ml.

Table S4: Fit parameters of LUV + MLV fit of native liposomes with 17 wt. ratio MBP aging series. Φ is the fraction of MLV. For the SLD of the MBP layer ρ_{MBP} no error is given since the fit error was extremely small. For the number of bilayers N, the Gaussian standard deviation of the number of bilayers dN and the thickness of the chain section d_{chain} no error is given since those values were fixed

	nat 0 days	nat 2 days	nat 4 days	nat 6 days	nat 8 days
Φ (%)	12.03±0.59	13.7 ±1.1	18.47 ±0.85	21.05±0.65	20.54±0.63
ρ_{MBP} (10^{-5} Å^{-2})	1.13	1.12	1.09	1.08	1.09
d_{MBP} (Å)	37	37	37	37	37
N	2	2	2	2	2
dN	0.5	1	1	1	1
d_{chain} (Å)	31.384 ± 0.050	31.078 ± 0.049	31.078 ± 0.049	31.078 ± 0.049	31.078 ± 0.049

Table S5: Fit parameters of LUV + MLV fit of EAE modified liposomes with 17 wt. ratio MBP aging series. Φ is the fraction of MLV. For the SLD of the MBP layer ρ_{MBP} no error is given since the fit error was extremely small. For the number of bilayers N, the Gaussian standard deviation of the number of bilayers dN and the thickness of the chain section d_{chain} no error is given since those values were fixed.

	EAE 0 days	EAE 2 days	EAE 4 days	EAE 6 days	EAE 8 days
Φ (%)	14.6±1.9	14.40 ±0.15	16.23 ±0.23	17.49±0.53	19.13±0.12
ρ_{MBP} (10^{-5} Å^{-2})	1.12	11.2	1.09	1.08	1.09
d_{MBP} (Å)	34	34	34	34	34
N	2	2	2	2	2
dN	0.5	0.5	0.5	0.5	0.5
d_{chain} (Å)	31	31	31	31	31

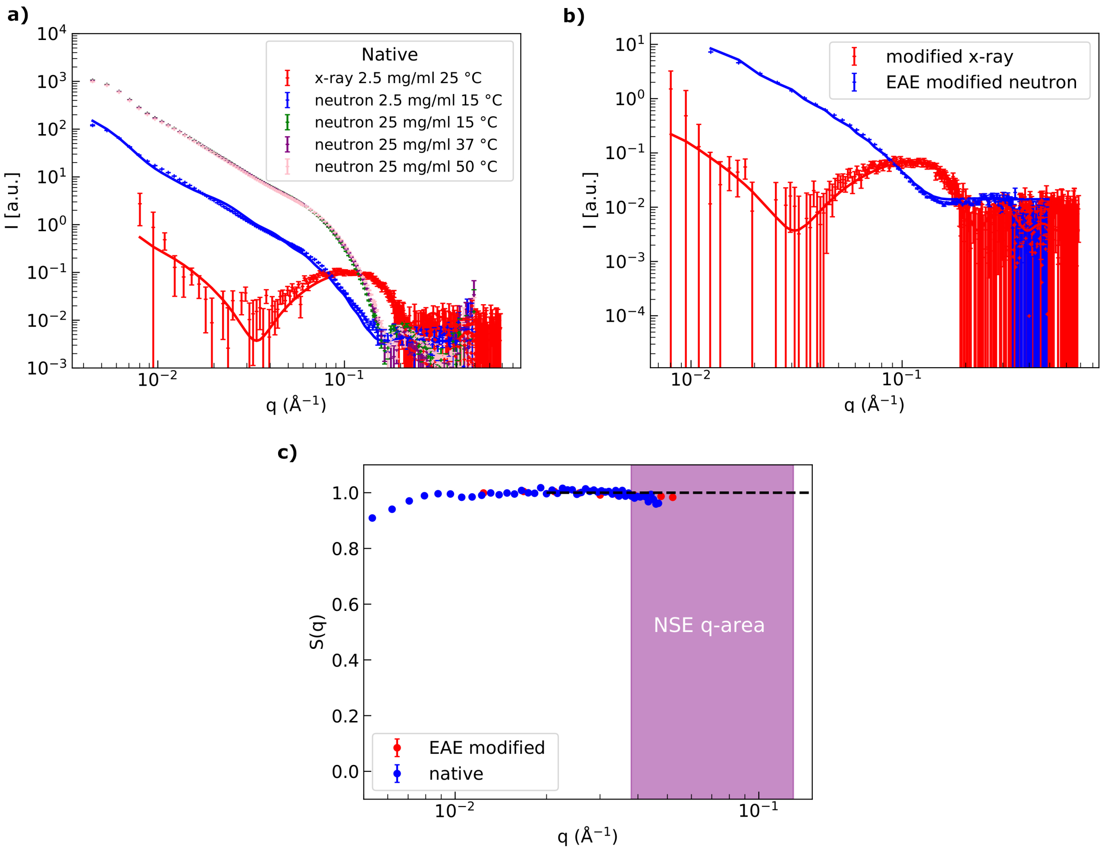

Figure S4: **a)** Native and **b)** modified SANS and SAXS data of NSE samples fitted with a simple LUV box-model. In **c)** the structure factor has been calculated from SANS data of the NSE samples with 25 mg/ml and diluted to 2.5 mg/ml.

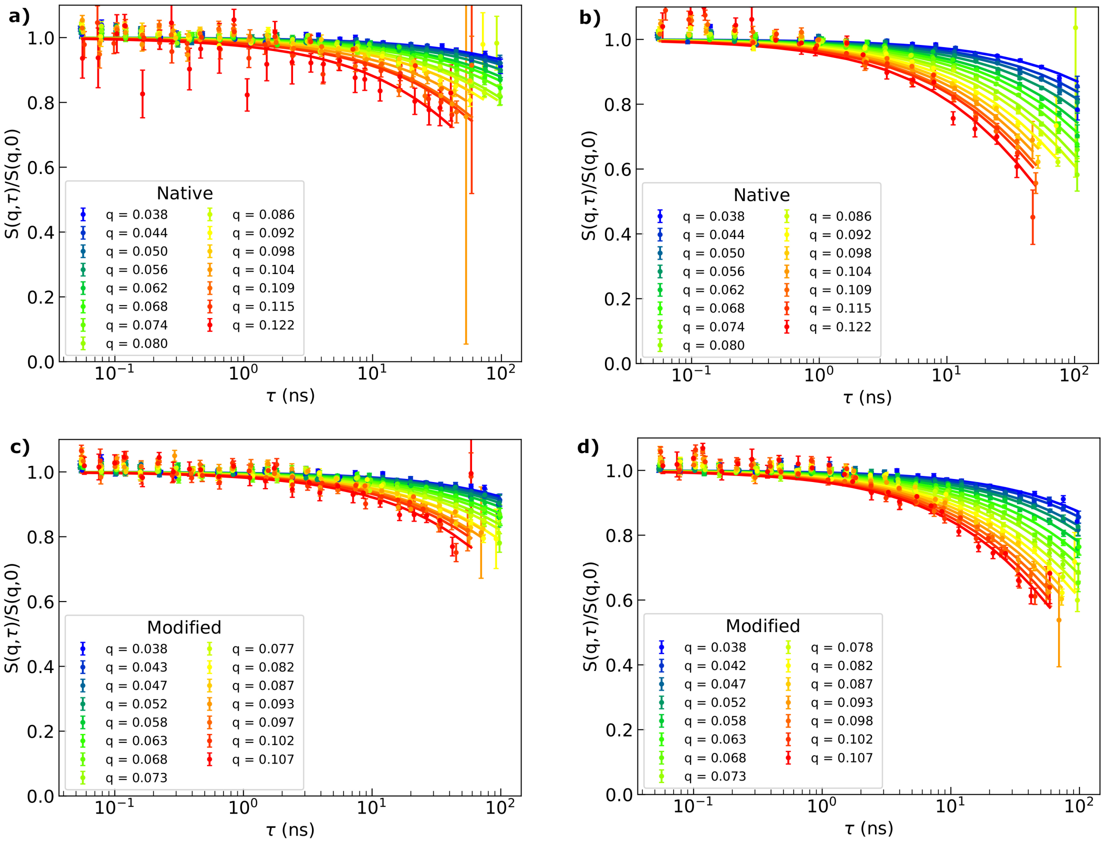

Figure S5: NSE data of native membranes at **a)** 10 °C and **b)** 37 °C and of modified membranes at **c)** 10 °C and **d)** 37 °C.

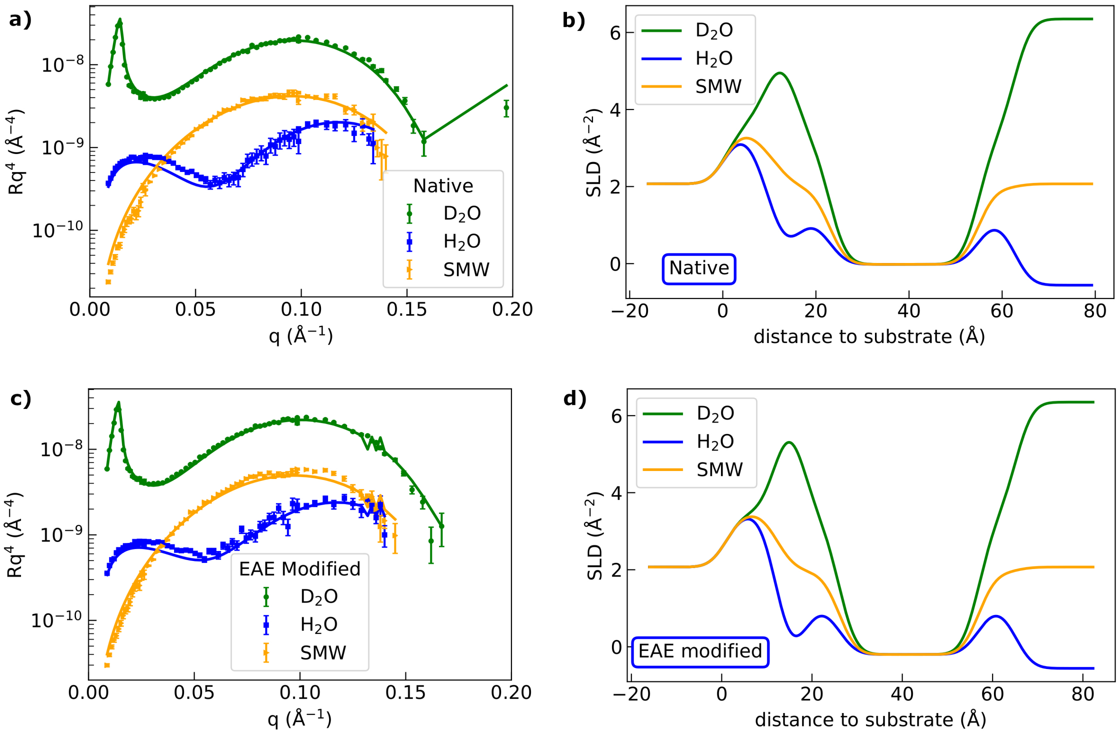

Figure S6: Neutron reflectivity curves of a) native and c) diseased myelin membranes with the respective fit to the stratified layer model with the layer order Si/SiOx/buffer/head/2·chains/head/buffer. The SLD profiles corresponding to the fits of the reflectivity curves of b) native and d) EAE modified myelin membranes.

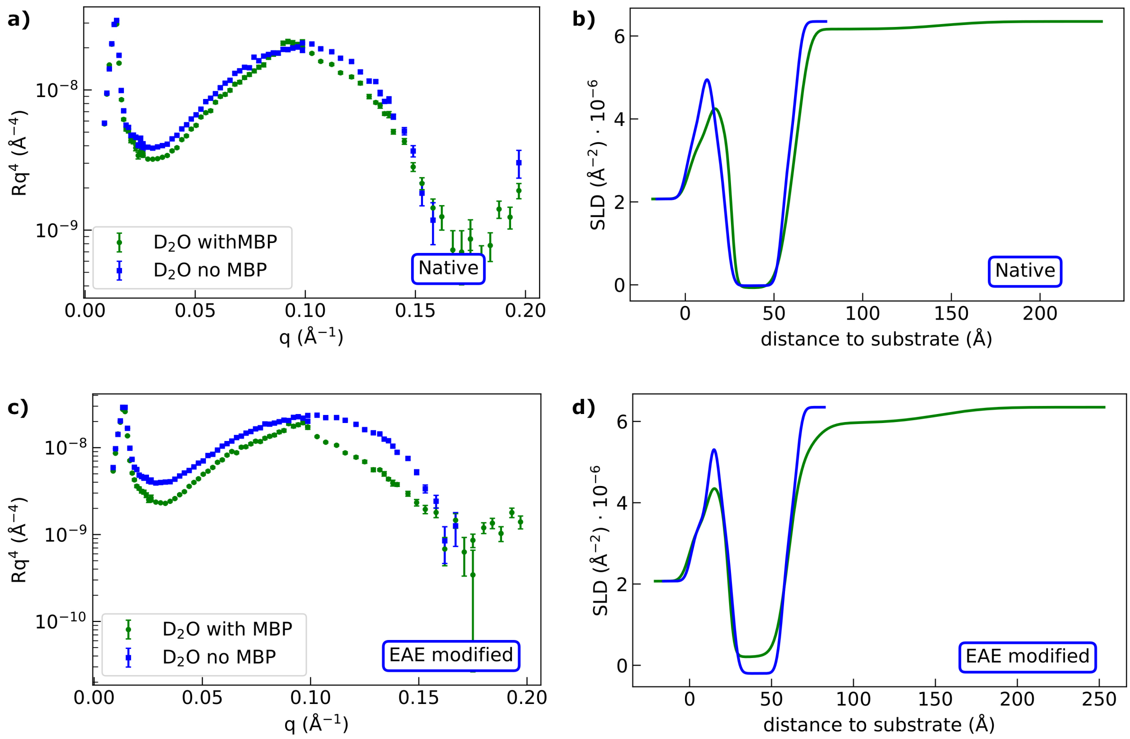

Figure S7: Comparison of reflectivity in D_2O of native and EAE modified b) membranes and their respective SLD plots (c) and d).

2 Materials and Methods

2.1 Materials

Porcine brain lipids (phosphatidylcholine, phosphatidylethanolamine, phosphatidylserine and sphingomyelin) and ovine cholesterol were purchased from Avanti Polar Lipids inc. (Alabaster, AL, USA). Bovine myelin basic protein (MBP) and all other chemicals were obtained commercially (Sigma-Aldrich, St. Louis, MO, USA).

2.2 Lipid Composition

In the scope of this work, two different types of liposomes have been prepared, which differ in their lipid composition. The exact compositions are shown in Tab. S6 and resemble native cytoplasmic myelin and EAE modified cytoplasmic myelin composition [2]. In the first step the different lipids are weighed with a total mass of 100 mg (Precisa XR 405A). The mixture is dissolved in 4 ml chloroform and distributed in 4 glass vials. The chloroform is then evaporated in the fume hood until full evaporation. Then the vials are left in a vacuum oven (Thermo Electron Corporation - Heraeus vacutherm) at 50 °C and around 10 mbar overnight. The lipid mix is afterwards either used immediately or stored at - 20 °C. The neutron and X-ray scattering length density (SLD) for the head and chain sections of the lipid mixtures is given in Tab. S7. The neutron (ρ_n) and X-ray (ρ_x) SLD values for the lipid mixtures are calculated from the lipid compositions as well as the chain section compositions of the different lipid types in Tab. S8 and the molecular volumes and scattering lengths of the chemical compounds found in lipid molecules in Tab. S9. The values of neutron scattering lengths for different isotopes and atomic form factors for X-rays can be found in literature [4, 5]. For the lipid compositions it has to be considered that the bovine chain composition distribution in myelin membranes might differ from the human one. It is possible that hygroscopic unsaturated lipids may pull some water out of the air during storage in the freezer and that the chain composition can change slightly.

2.3 Liposome Preparation

The liposomes of composition as shown in Tab. S6 were prepared following an extrusion protocol. In a first step they were hydrated with buffer (10 mM 3-(N-morpholino)propanesulfonic acid (MOPS), 150 mM sodium

Table S6: Native χ_{nat} and EAE modified χ_{EAE} molar lipid fractions. Values and structure taken from [2, 3]. For the phospholipids PC, PE, PS and SM only an exemplary structure is shown, since there is a broad distribution of fatty acid chains in myelin membranes.

lipid type	χ_{nat} (%)	χ_{EAE} (%)
Phosphatidylcholine 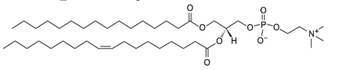	25.9	20.1
Phosphatidylethanolamine 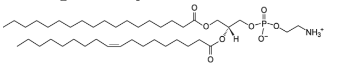	29.0	32.9
Phosphatidylserine 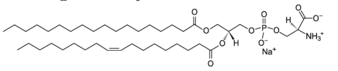	7.0	7.4
Sphingomyelin 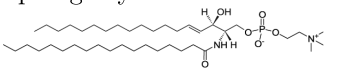	6.2	2.2
Cholesterol 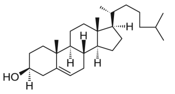	31.6	37.4

Table S7: SLD values of head and chain sections of native and EAE modified lipid compositions. The values for X-rays were calculated for 9.3 keV and 12 keV photon energy. The difference is smaller than 1% and therefore negligible compared to uncertainties in preparation and composition. The errors for the chain SLD arise from the unknown fraction of the fatty acid distribution (Tab. S8).

lipid	ρ_{n} ($\text{Å}^{-2} \cdot 10^{-6}$)	ρ_{x} ($\text{Å}^{-2} \cdot 10^{-6}$)
native head	1.87	14.2
EAE head	1.83	14.6
native chain	-0.071 ± 0.04	8.3 ± 0.4
EAE chain	-0.059 ± 0.03	8.4 ± 0.4

chloride (NaCl), pH/ pD 7.0). The solution was shaken for 30 minutes at 50 °C. In the case that some lipids still remained on the glass surface afterwards, they were removed by pipetting the solution several times. In the following, the liposome solution was sonicated for 30 min at 50 °C (followed by 3 freeze thaw cycles with liquid nitrogen). To filter out big particles, which impede an extrusion, the solutions were filtered through a 0.45 µm centrifugal filter for 10-15 minutes at (10.000 – 13.000) g at 40 °C. Afterwards the solution was extruded 19 or 21 times with an Avanti Mini Extruder using a polycarbonate membrane with 100 nm pore size. The temperature was kept at 50 °C during the extrusion with a heating block on a IKA RCT basic heating plate. For SANS experiments deuterium oxide was used as buffer solvent.

Table S8: fatty chain compositon of different lipid types taken from [3].

Fatty acid type	PC (%)	PE (%)	PS (%)	SM (%)
14:0	0.3	0	0	0
16:0	30.6	3.7	0	2
16:1	0.9	0.6	0	0
18:0	16.5	15.9	42	50
18:1	33.3	24.1	30	0
18:2	1.1	0.4	0	0
20:0	0	0	0	5
20:1	0.8	2.3	0	0
20.2	0	0.6	0	0
20:3	0.2	0.6	0	0
20:4	3.1	18.6	2	0
22:0	0	0	0	7
22:1	0	0.5	0	0
22:6	0.6	11.5	11	0
24:0	0	0	0	5
24:1	0	0	0	21
unknown	12.8	21.1	15	10

Table S9: Molar volumes, coherent scattering lengths, neutron (ρ_n) and 12.61 keV x-ray (ρ_x) scattering length density of different lipid head groups and carbohydrates. Molar volumes for carbohydrate and lipid groups were taken from [a] [6], [b] [7], [c] [8] and [d] [9]. Molar volumes for carbohydrate groups were taken from a measurement of DOPC. ρ_n was calculated from the atomic scattering lengths from [4]. ρ_x was calculated from the atomic form factors [10].

lipid	V_m (Å^3)	b_coh (fm)	ρ_n (Å$^{-2} \cdot 10^{-6}$)	ρ_x (Å$^{-2} \cdot 10^{-6}$)
PC head	346.7^a	60.07	1.73	12.7
PE head	312^b	62.56	2.01	16.3
PS head	278.7^a	77.90	2.80	13.4
SM head	323.8^c	62.56	1.93	12.3
Cholesterol	629^d	13.31	0.21	9.7
CH3	51.9^a	−4.57	-0.88	2.2
CH2	27.2^a	−0.83	-0.31	3.1
CH	22.6^a	2.91	1.29	2.5

2.4 Small-Angle X-Ray Scattering

SAXS was measured on the SWING beamline at SOLEIL synchrotron (Saint-Aubin, France) [11]. The used photon energy was 12 keV$\hat{=}$ 1.0332 Å X-ray wavelength, and the detector-sample distance was 3.37 m. The X-ray detector of SWING is a EIGER 4M. In addition, we performed SAXS experiments on the in-house instrument GANESHA (SAXSLAB) located at JCNS-1. The GANESHA setup uses a liquid metal-jet source with Gallium kα x-rays with a wavelength of λ =1.3414 Å, and a detector-sample distance of 0.57 m covering a q-range from 0.015 to 0.3 Å^{-1}. The x-ray detector of the Ganesha beamline is a Pilatus 300k. The temperature was kept at room temperature (26 °C) for all SAXS experiments. Prior to subtraction of the background all curves were radially averaged, scaled to measurement time, transmission and sample thickness. Liposomes of native and EAE modified lipid composition were prepared as described above. Liposomes with lipid concentration of 5 mg/ml were measured on SWING. MBP with concentration of 0.5 mg/ml was added. On GANESHA liposomes at concentration of 10 mg/ml and liposomes with 5 mg/ml plus 0.5, 1, 2 and 5 mg/ml MBP were investigated. Fitting of SAXS and also SANS data has been performed with the python based software jscatter [1].

2.5 Small-Angle Neutron Scattering

Measurements were performed on KWS-2 operated by Juelich Centre for Neutron Science (JCNS) at Heinz Maier-Leibnitz Zentrum (MLZ) in Garching [12]. Liposomes of native lipid composition and EAE modified lipid composition were prepared as described above. The liposome concentration was 2.5 mg/ml and the MBP concentration 0.5 mg/ml. The temperature was room temperature. Sample volumes of 300 μL were filled into Hellma 1 mm quatz glass cuvettes. These were measured with the automatic sample exchange system of the KWS-2 instrument. The following instrument configurations were used for the measurements: Wavelenght and

Sample-to-detector distances of $\lambda = 5\,\text{Å}$ at 2 m, 4 m and 8 m, $\lambda = 10\,\text{Å}$ at 20 m, $\lambda = 7\,\text{Å}$ at 16 m with focusing lenses to cover a maximum q-range from $1.05 \cdot 10^{-4}$ to $4.6 \cdot 10^{-1}$ Å. The data treatment was performed using the QtiKWS program [13] which issue the scattering cross section of the measured sample in absolute unit after subtracting empty cell, empty bean, and dark current from a Boron Carbide measurement as well as the buffer. The data are normalization to a Plexiglass (Poly-methyl-methacrylate) standard sample

2.6 Cryo-TEM

Samples for TEM were prepared by placing a drop of 5 µl solution on a lacey S7/2 carbon-coated copper grid. The grids were glow discharged for 30 seconds at 8 mA in Argon atmosphere prior to the preparation. After a few seconds, excess solution was removed by blotting with filter paper. This sample was cryo-fixed by rapid immersion into liquid ethane at -180 °C using a cryo-plunger (EMGP Leica GmbH). Temperature was set to 20 °C and the relative humidity at 80% in the cryo-plunger prior to vitrification. The specimen was inserted into a cryo-transfer holder (HTTC 910, Gatan, Munich, Germany) and transferred to a JEM 2200 FS EFTEM instrument (JEOL, Tokyo, Japan). Examinations were carried out at temperatures around -180 °C. The transmission electron microscope was operated at an acceleration voltage of 200 kV. Zero-loss electron energy filtered images were taken under reduced dose conditions ($< 10000\,e^-/nm^2$). All images were recorded digitally by a bottom-mounted 16 bit CMOS camera system (TemCam-F216, TVIPS, Munich, Germany). To avoid any saturation of the gray values, all the measurements were taken with intensity below 15 000, considering that the maximum value for a 16 bit camera is 2^{16}. Images have been taken with EMenu 4.0 image acquisition program (TVIPS, Munich, Germany) and processed with a free digital imaging processing system Image J [14–16].

2.7 Neutron Reflectometry

Neutron reflectivity data were acquired on the MARIA reflectometer [17] operated by JCNS at MLZ in Garching (Germany), using custom temperature-regulated liquid cells. The measurements were performed using two different wavelengths, 10 Å for the low-q region and 5 Å for the high-q region up to $0.25\,\text{Å}^{-1}$, with a wavelength spread of 0.1. The change of solvent contrast in the liquid cells was performed using a combination of valves and a peristaltic pump, at small flow rates of 0.5 ml/min. All fits were done with the IGOR Pro based software motofit [18]. As the fitting algorithm of motofit is on stochastic nature, different runs lead to slightly different values for each fitted parameter. Therefore, the errors of the fits of the pure membranes were estimated by performing multiple fitting runs. For the membranes with MBP these differences of the results are more significant as the MBP layer is dilute and highly hydrated. Hence, the fit was repeated 10 times and from the respective fit results we calculated the mean values for the fit parameters and the standard deviations as errors. This allows both error estimation and an evaluation of the stability of the fits.

2.8 NR sample preparation

Two Andreas-Holm GmbH ultra-polished Si blocks (r.m.s. roughness 1-2 Å, dimensions 150x50x20 mm) were cleaned with water and ethanol and treated with an UV-ozone plasma etching (Novascan technologies) for 10 minutes 2 times. Then the liquid cells were assembled immediately and H_2O buffer was injected in the cell. The liquid cell is made of boronglas and sealed by a Viton o-ring. This kind of cell has already been described in [19]. After alignment of the sample in respect of the beam the thickness and roughness of the Silicon oxide layer was characterized. In one sample liposomes with native lipid composition were injected and in the other one liposomes with EAE modified lipid composition. The liposomes were prepared in H_2O-buffer with the extrusion protocol described above and had a concentration of 1 mg/ml. The vesicles were kept for 10 minutes at 50 °C to fuse on the substrate. Afterwards the remaining liposomes were flushed out with buffer. Now the samples were measured first at H_2O buffer, then in silicon-matched-water (SMW)-buffer with a D2O-ratio of 38% and third in D_2O-buffer. To check at which concentration of MBP a significant change of structure of the native membrane can be observed, we injected MBP of increasing concentration in D_2O-buffer starting at 1 µg/ml and time resolved neutron reflectometry was measured. As for 100 µg/ml a significant change was observed, we also injected this concentration to the modified membrane and again measured neutron reflectivity at D_2O, SMW and H_2O contrast.

2.9 Neutron Spin Echo Spectroscopy

The NSE experiment was performed at the SNS-NSE spectrometer [20] at the Oak Ridge National Laboratory. Measurements were carried out combining incident wavelength from 8 Å to 11 Å for minimum momentum transfer between $q = 0.035$ and $q = 0.13\,\text{Å}^{-1}$. The NSE spectra were collected at 10 °C - 50 °C for samples in 4 mm-path quartz cells, accessing a dynamical range between $0.1 \leqslant \tau_{\text{max}} \leqslant 130ns$. Al2O3 and Graphite foil were used as standard elastic reference and D2O solvent measurements in identical sample holder were also necessary for proper data reduction. Liposomes of both lipid compositions were measured in a concentration of 25 mg/ml. The unilamellarity, vesicle structure factor and temperature dependence of the vesicles are probed by measuring SANS and SAXS. The data reduction was performed with the standard software package of the SNS-NSE instrument called DrSpine [21]. Since SNS-NSE is a time-of -flight technique the data were acquired with enough statistics so the entire wavelength spectra can be grouped in 20 different solid angles (or how many q-values you got) up to a maximum $q = 0.25\,\text{Å}^{-1}$ for a better discretization of the q-dependence.

3 Small Angle Scattering Fitting Models

3.1 LUV model

The vesicles without MBP can be fitted by a simple liposome box model. The layer sequence is buffer/lipid heads/alkyl chains/lipid heads/buffer. The scattering intensity $I_{\text{luv}}(q)$ can be written as:

$$I_{\text{luv}}(q) = \Phi V_{\text{outershell}} F(q), \tag{1}$$

with the volume fraction Φ, the volume of the outer shell $V_{\text{outershell}}$ and the form factor $F(q)$:

$$F(q) = \left(\sum_i \rho_{\text{i}} d_{\text{i}} F_{\text{S}}(q, R_{\text{i}}) \right)^2 . \tag{2}$$

Here, the membrane head and chain thickness d_{i} are fixed to the values obtained from the neutron reflectometry (NR) data of the oriented bilayer. The scattering length density of the respective layer ρ_{i}.

$$\rho_{\text{i}} = \rho_{\text{comp,i}} h \tag{3}$$

is calculated by the product of the SLD of the calculated layer composition, see Tab. S7, and the layer hydration h. F_{S} is the form factor of a sphere:

$$F_{\text{S}} = 3\frac{\sin(qR) - qR\cos(qR)}{(qR)^3}, \tag{4}$$

with sphere radius R and scattering vector q.

3.2 MLV model

After addition of MBP to the liposomes in addition to unilamellar vesicles, multilamellar structures can be found in the cryo-TEM. Therefore, we model the SAS-data - which was normalized to the intensity distance between the local minimum at $q \sim 0.1\,\text{Å}^{-1}$ and the background at $q \geq 0.3\,\text{Å}^{-1}$ - by adding a multilamellar vesicle fraction with the intensity I_{MLV} to the unilamellar vesicle form factor I_{LUV}. The two constituents are weighted by a ratio factor ϕ and the sum is multiplied again by a scaling factor A. Additionally a background c is added.

$$I(q) = c + A \cdot [(1-\phi) I_{\text{LUV}}(q) + \phi I_{\text{MLV}}] . \tag{5}$$

I_{MLV} is calculated by a spherical multi-shell model with the form factor, which can be described as Eq. 2 with the difference that in the form-factor F_{MLV} additional layers are added.

$$I_{\text{MLV}} = V_{\text{outershell}} F_{\text{MLV}}(q), \tag{6}$$

The order of the shells is chosen to be buffer + [head,2 · chains,head] + (N-1)*[MBP,head,2 · chains,head]. The SAXS scattering intensity is calculated like in Eq. 1. In this model we added polydispersity to the inner radius R_{MLV} of the MLV and to the number of bilayers N by calculating the intensity for different values and weighting them with a normalized Gaussian with $\sigma = dR, dN$. In the case of N only integer values >= 2 are calculated. The parameters of the LUV, the thickness, the SLD and hydration of head and chain section in the MLV part were fixed to the values of the corresponding LUV fit. Only the thickness of the native head section had to be varied in the error limits of the thickness calculated from the NR data. As the q-values we probe in the SAXS experiments give only insights to the local structure of our sample the inner radius and its polydispersity is fixed

to the same constant value for all curves. The mean number of bilayers $N = 2$ is known from the cryo-TEM as we see in most images vesicles sticking together (Fig. S2). The polydispersity dN which allows to introduce higher number of bilayers in our model is fixed to $dN = 0.5$ as the peak shape does not change with the concentration. Only in the native aging data the shape of the peak changes. Therefore, we need to increase dN in this case. The thickness of the MBP layer between the bilayers changes the position of the peaks. Therefore, we set d_{MBP} by the peak position. Finally, only the scaling factor A, the ratio of MLV Φ and the SLD of the MBP layer ρ_{SLD} are fitted.

References

[1] R. Biehl, *Jscatter, a Program for Evaluation and Analysis of Experimental Data*, Available at https://pypi.org/project/jscatt https://gitlab.com/biehl/jscatter, Mar. 2019.

[2] Y. Min, K. Kristiansen, J. M. Boggs, C. Husted, J. A. Zasadzinski, and J. Israelachvili, "Interaction forces and adhesion of supported myelin lipid bilayers modulated by myelin basic protein", Proceedings of the National Academy of Sciences **106**, 3154–3159 (2009).

[3] *Avanti polar lipids, inc.* https://avantilipids.com, Accessed: 2018-10-11.

[4] V. F. Sears, "Neutron scattering lengths and cross sections", Neutron news **3**, 26–37 (1992).

[5] B. Henke, E. Gullikson, and J. Davis, "Xray interactions: photoabsorption, scattering, transmission, and reflection at e = 50-30,000 ev, z = 1-92", Atomic Data and Nuclear Data Tables **54**, 181–342 (1993).

[6] J. C. Fogarty, M. Arjunwadkar, S. A. Pandit, and J. Pan, "Atomically detailed lipid bilayer models for the interpretation of small angle neutron and x-ray scattering data", Biochimica et Biophysica Acta (BBA) - Biomembranes **1848**, 662–672 (2015).

[7] S. Leikin, M. M. Kozlov, N. L. Fuller, and R. P. Rand, "Measured effects of diacylglycerol on structural and elastic properties of phospholipid membranes", Biophysical journal **71**, 2623–2632 (1996).

[8] P. Niemelä, M. T. Hyvönen, and I. Vattulainen, "Structure and dynamics of sphingomyelin bilayer: insight gained through systematic comparison to phosphatidylcholine", Biophysical Journal **87**, 2976–2989 (2004).

[9] J. Pencer, T. Mills, V. Anghel, S. Krueger, R. M. Epand, and J. Katsaras, "Detection of submicron-sized raft-like domains in membranes by small-angle neutron scattering", The European Physical Journal E **18**, 447–458 (2005).

[10] C. T. Chantler, "Theoretical form factor, attenuation, and scattering tabulation for z=1–92 from e=1–10 ev to e=0.4–1.0 mev", Journal of Physical and Chemical Reference Data **24**, 71–643 (1995).

[11] G. David and J. Pérez, "Combined sampler robot and high-performance liquid chromatography: a fully automated system for biological small-angle X-ray scattering experiments at the Synchrotron SOLEIL SWING beamline", Journal of Applied Crystallography **42**, 892–900 (2009).

[12] A. Radulescu, N. K. Szekely, and M.-S. Appavou, "Kws-2: small angle scattering diffractometer", Journal of large-scale research facilities JLSRF **1**, 29 (2015).

[13] V. Pipich, *Qtikws: user-friendly program for reduction, visualization, analysis and fit of sa (n) s data*, 2012.

[14] W. Rasband, "Imagej: image processing and analysis in java", Astrophysics Source Code Library (2012).

[15] C. A. Schneider, W. S. Rasband, and K. W. Eliceiri, "Nih image to imagej: 25 years of image analysis", Nature methods **9**, 671 (2012).

[16] M. D. Abràmoff, P. J. Magalhães, and S. J. Ram, "Image processing with imagej", Biophotonics international **11**, 36–42 (2004).

[17] S. Mattauch, A. Koutsioubas, U. Rücker, D. Korolkov, V. Fracassi, J. Daemen, R. Schmitz, K. Bussmann, F. Suxdorf, M. Wagener, P. Kämmerling, H. Kleines, L. Fleischhauer-Fuß, M. Bednareck, V. Ossoviy, A. Nebel, P. Stronciwilk, S. Staringer, M. Gödel, A. Richter, H. Kusche, T. Kohnke, A. Ioffe, E. Babcock, Z. Salhi, and T. Bruckel, "The high-intensity reflectometer of the Jülich Centre for Neutron Science: MARIA", Journal of Applied Crystallography **51**, 646–654 (2018).

[18] A. Nelson, "Co-refinement of multiple-contrast neutron/X-ray reflectivity data using *MOTOFIT*", Journal of Applied Crystallography **39**, 273–276 (2006).

[19] A. Koutsioubas, "Combined coarse-grained molecular dynamics and neutron reflectivity characterization of supported lipid membranes", The Journal of Physical Chemistry B **120**, PMID: 27748120, 11474–11483 (2016).

[20] M. Ohl, M. Monkenbusch, N. Arend, T. Kozielewski, G. Vehres, C. Tiemann, M. Butzek, H. Soltner, U. Giesen, R. Achten, H. Stelzer, B. Lindenau, A. Budwig, H. Kleines, M. Drochner, P. Kaemmerling, M. Wagener, R. Moeller, E. Iverson, M. Sharp, and D. Richter, "The spin-echo spectrometer at the spallation neutron source (sns)", English, Nuclear Instruments and Methods In Physics Research **696**, 85–99 (2012).

[21]P. Zolnierczuk, O. Holderer, S. Pasini, T. Kozielewski, L. Stingaciu, and M. Monkenbusch, "Efficient data extraction from neutron time-of-flight spin-echo raw data", Journal of applied crystallography **52** (2019).
